# Gender Inequities in the Impact of Climate Change on Health: A Scoping Review

**DOI:** 10.3390/ijerph21081093

**Published:** 2024-08-19

**Authors:** Melina Denise Zavala, Cintia Cejas, Adolfo Rubinstein, Analia Lopez

**Affiliations:** Center for Implementation and Innovation in Health Policies, Institute for Clinical Effectiveness and Health Policy, Buenos Aires C1414CPV, Argentina; ccejas@iecs.org.ar (C.C.); arubinstein@iecs.org.ar (A.R.); alopez@iecs.org.ar (A.L.)

**Keywords:** climate change, health and well-being, gender, social determinants of health, inequities

## Abstract

In the 21st century, climate change has emerged as a critical global public health challenge. Women experience the most severe impacts of climate change, intensifying pre-existing gender inequalities. This scoping review aims to explore the intersection of climate change, health, and gender, considering the social determinants of health. The methods for this review follow the Arksey and O’Malley framework for a scoping review and the PRISMA-ScR checklist. The review, covering January 2019 to February 2024, included PubMed, LILACS, and SciELO databases. We identified 71 studies with 19 meeting the inclusion criteria. The results revealed the differential effects of climate change on health according to gender in areas such as mental health, reproductive health, gender-based violence, occupational health, and health issues associated with heat and air pollution. Our findings also elucidated how socio-economic and gender inequities intersect, exacerbating the risk of experiencing these effects. In conclusion, the study highlights a clear need for gender-sensitive climate policies and interventions to address these disparities and protect vulnerable populations from the health impacts of climate change.

## 1. Introduction

In the 21st century, climate change poses one of the greatest challenges to global public health, as unprecedented warming impacts all dimensions of life, including health. 

Its effects are both direct, through heat waves, droughts, and strong storms, and indirect, through exacerbating diseases and injuries, including respiratory and cardiovascular diseases, strokes, food- and water-borne diseases, vector-borne diseases, and mental health disorders, among many others [1,2]. These health problems are affected by environmental conditions to the extent that populations experience adverse social, political, economic, environmental, and cultural conditions that constitute vulnerabilities [3].

Although we are all exposed to climate hazards or threats, the impact of adverse climatic conditions affects populations differently. In addition to variations in probability and intensity of exposure to hazards, populations also have varying capacities to respond to or anticipate adverse climate events [4].

According to the United Nations, the climate crisis does not affect all genders equally [5]. Women and girls experience the most severe impacts of climate change, intensifying pre-existing gender inequalities and posing particular threats to their ways of life, health, and safety. 

Women who are socially and economically disadvantaged, including those who are young, poor, poorly educated, and/or live in rural areas, have the greatest difficulty in obtaining the health services they need. In addition, highly vulnerable populations comprising systematically marginalized groups such as indigenous populations; people of African descent; Lesbian, Gay, Bisexual, Trans, Queer, and Intersex (LGBTQI+) people; people with disabilities; and migrants, among others, often face discrimination and stigmatization; they face the greatest structural barriers to accessing health services and ultimately suffer the worst health outcomes. All of these conditions of marginality intensify when they coexist and intersect with each other [1,6].

In the face of the climate crisis, vulnerable populations are more likely to suffer negative impacts, ranging from extreme weather events to the loss of essential resources and mass displacement, among others. In this context, the role of the health sector in protecting communities by ensuring access to services and highlighting the importance of healthy environments for human well-being is crucial.

In recent years, there has been significant progress in integrating a gender perspective into the planification and implementation of climate action at the national, regional, and global levels. This progress has been driven by the explicit inclusion of gender dimensions in a series of national climate commitments set by the signatory countries under the Paris Agreement of 2015. This important milestone reflects the growing recognition of the intersection between health, gender, and climate change within the 2030 Agenda and the Sustainable Development Goals (SDGs) [7].

While research on climate change’s health impacts exists, the current literature predominantly focuses on specific climatic factors rather than offering a comprehensive view. Additionally, few studies delve into understanding the issue from a gender perspective. 

A scoping review offers a comprehensive overview through a systematic approach, allowing for the survey the evidence on a topic and the identification of key concepts, theories, sources, and knowledge gaps. This method enables a thorough exploration of the existing literature, providing valuable insights into the intricacies of the subject matter [8].

This scoping review aims to fill a gap by exploring how climate change, human health, and gender intersect, considering the social determinants of health. Ultimately, we seek to contribute to a comprehensive understanding of this intersection, thereby guiding the design of gender-sensitive interventions and policies.

## 2. Materials and Methods

This scoping review followed the framework proposed by Arksey and O’Malley [9]. This methodology is composed of five consecutive stages: (1) identification of the research question, (2) identification of relevant studies, (3) selection of studies, (4) data extraction, and (5) collation, summarization, and communication of results. Each stage is discussed below.

Additionally, this review adhered to the Preferred Reporting Items for Systematic reviews and Meta-Analyses extension for Scoping Reviews (PRISMA-ScR) checklist [8].

### 2.1. Phase 1: Identification of the Research Question

Arksey and O’Malley suggest an iterative process for developing one or more research questions. The following research questions guided the present study:

- What are the differentiated impacts of climate change on health considering the gender dimensions?
- How are socioeconomic and gender inequalities intertwined in exposure to climate risks?

### 2.2. Phase 2: Identification of Relevant Studies

To identify the relevant literature, a search was carried out in Spanish and English in the following databases: PubMed, LILACS, and SciELO, with a specific search strategy. These three databases were selected for their relevance and accessibility. PubMed provides extensive coverage of the biomedical and health-related literature, making it essential for health research. To address the underrepresentation of the Latin America and Caribbean region in global research, we also included SciELO and LILACS to complement our search. 

The search criteria and terms were developed in collaboration with an institutional documentalist/librarian to ensure an exhaustive and comprehensive search.

The combination of search terms used can be found in Table 1.

### 2.3. Phase 3: Study Selection

Inclusion criteria for the review encompassed systematic reviews, meta-analyses, or scoping reviews published in either Spanish or English between January 2019 and February 2024, addressing the intersection of climate change, health, and gender. There were no restrictions on the study periods of the articles, allowing the inclusion of studies examining time periods longer than five years. This approach enabled the capture of broader trends and foundational studies in the field. 

The selection of this time period was influenced by the fact that, while there is extensive literature on the relationship between climate and health, the intersection of gender studies with climate and health is relatively recent, and the body of published research in this specific area is limited. 

The decision to focus exclusively on reviews was made for two main reasons. Firstly, reviews provide a comprehensive synthesis of the existing literature, allowing for a deeper understanding of trends, findings, and consensus within the field. Secondly, reviews facilitate the identification of emerging trends and areas of agreement or divergence across studies, which is crucial for guiding future research directions.

Exclusion criteria comprised studies focused solely on animal populations or lacking an examination of the relationship between climate change, health, and gender. Additionally, studies with a broad scope, either at the global or regional level, were eligible for inclusion, while those specific to a particular country were excluded from the analysis.

The selection process for indexed studies was carried out in two stages. The first consisted of the screening of each title and abstract in COVIDENCE by two reviewers independently to determine its eligibility for full text selection. Each article was classified into one of three categories (Yes, Maybe, No) to assess the relevance and likelihood of full-text retrieval. In the second phase, all articles except those categorized as “No” (excluded) were retrieved in full text for further analysis. Disagreements between reviewers were resolved by consensus.

### 2.4. Phase 4: Data Extraction

Data extraction was performed using a qualitative descriptive approach. Each article was extracted manually. The information surveyed was charted in an Excel sheet and categorized based on author and year of publication; type of article; region; study period; number of articles included in the review; aim; population analyzed; main health factor identified; environmental factor identified; and key findings. 

### 2.5. Phase 5: Summary and Reporting of Results

A synthesis of the collected data was conducted using thematic analysis, allowing for a narrative integration of the reviewed articles and the identification of common themes. The key findings in the literature were summarized in tabular form, providing a clear and organized overview of critical aspects. The results of this analysis were presented narratively, highlighting the emerging connections and patterns.

## 3. Results

**Figure 1:**
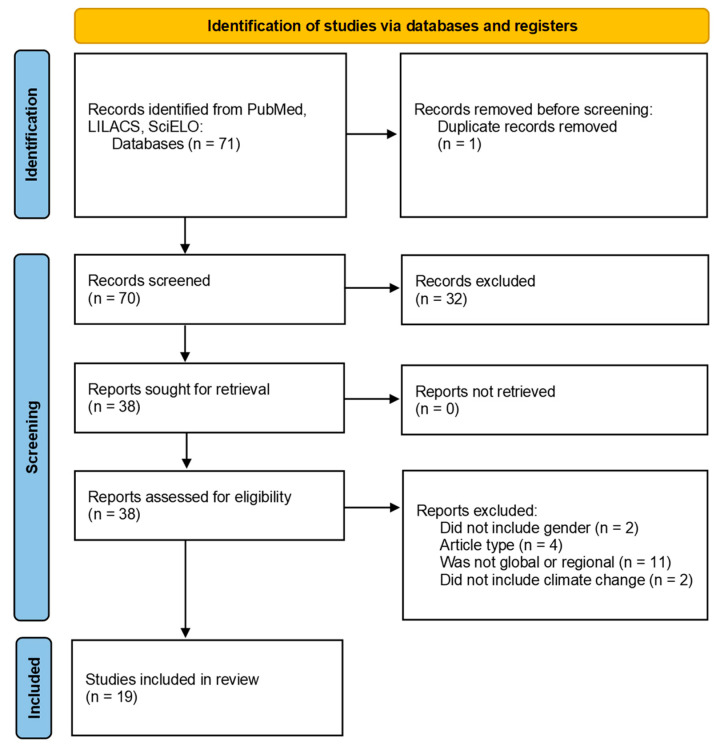
PRISMA flow diagram.

A total of 71 articles were identified, of which 33 (46%) were identified in PubMed, 23 (32%) in LILACS, and 15 (21%) in SciELO. After removing duplicates, 70 articles were included for the screening of titles and abstracts. In this screening, 32 studies were excluded, and the remaining 38 were assessed for eligibility by reviewing the full text. Of these 38 articles, 19 were included. A total of 11 were excluded because they were country-specific studies, 4 because of the study type, 2 because they did not include climate change aspects, and 2 because they did not refer to gender (Figure 1). The study characteristics are presented in Table 2.

### 3.1. Themes

Of the 19 included articles, five themes were developed through thematic analysis: mental health, reproductive health, gender-based violence, occupational health implications, and other health issues associated with heat and air pollution. A summary of the key results is shown in Table 3.

**Figure 2:**
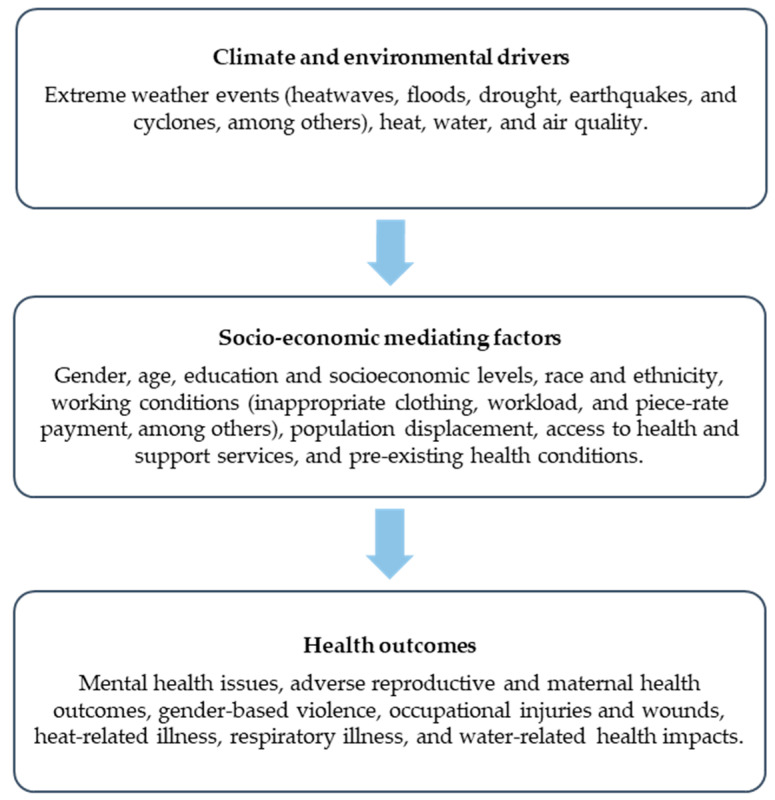
Flow diagram of climate and environmental drivers, socio-economic mediating factors, and health outcomes.

Figure 2 illustrates how climate and environmental factors lead to various health effects, which are mediated by the social and economic conditions in which individuals live and develop.

#### 3.1.1. Mental Health

The global burden of mental health conditions is already considerable, even without taking into account the effects of climate change [29]. The latter has shown impacts ranging from adverse emotional reactions, such as guilt, sadness, and discouragement, to more serious conditions such as anxiety, depression, and post-traumatic stress disorder, as well as suicidal ideation and intentions [25,30,31].

Chique et al. [25] have reported that women face a higher risk of psychological impairment after extreme weather events, especially showing increased rates of post-traumatic stress disorder (PTSD) and anxiety. Using floods as an example, Stone et al. [11] illustrate that women experience anxiety not only concerning their own health but also regarding their children’s health and safety due to the increased risk of falling into streams or contracting water-borne diseases. Additionally, women show concern over financial hardships caused by climate change, as well as sadness over the inability to access land resources, among other negative emotions.

The increasing visibility of the effects of climate change on mental health has expanded the concept of eco-anxiety, defined by the American Psychological Association as “a chronic fear of environmental doom” [32].

Rothschild and Haase [27] examine how eco-anxiety has influenced women’s reproductive health decisions. This has led some women to decide against having children due to environmental concerns. This reflects that the connection between climate change and reproductive health not only affects pregnant women physically but also their psychological well-being. In that sense, Huang et al. [13] highlight that pregnant women face a substantially higher risk of PTSD after exposure to cyclones, persisting for up to two years post-exposure.

Concerning children’s mental health, Adu et al. [21] demonstrate how various sociodemographic factors, including gender, are associated with or contribute to the type, prevalence, and severity of children’s psychological reactions after wildfires.

#### 3.1.2. Reproductive Health

In the literature reviewed, there is a growing body of evidence linking climate change to adverse reproductive health outcomes. 

In the Eastern Mediterranean region and the Middle East region, Neira et al. [20] conducted a review exploring health risks associated with extreme heat, water scarcity, air pollution, vector-borne diseases, and the health challenges faced by displaced populations. Their review highlighted the significant adverse effects of extreme heat exposure, particularly on pregnant women and their unborn children, such as complications during pregnancy and mortality related to malaria. In addition, displacement poses heightened risks for pregnant women, stemming from inadequate hygiene, nutrition, and access to antenatal and postnatal care. These challenges, including reproductive tract infections, malnourishment, and limited medical services, exacerbate the vulnerability to maternal morbidity and mortality, preterm birth, miscarriage, stillbirth, and other perinatal and neonatal complications.

Pregnant women also face challenges related to workplace conditions and environmental exposures. Rekha et al. [16] conducted a comprehensive review exploring the association between elevated workplace/ambient temperatures and adverse pregnancy outcomes, including miscarriages, preterm births, stillbirths, low birth weight, and congenital anomalies. The review’s findings highlight the need of epidemiological research to prevent occupational heat-related diseases, especially on vulnerable working pregnant women in developing countries.

Pregnant women are particularly vulnerable to increasing ambient temperatures and heat waves due to their compromised ability to thermoregulate [33]. These high temperatures frequently coincide with droughts. Droughts, defined as prolonged periods of dryness marked by a significant deficiency in precipitation, are complex phenomena that develop over time and exert profound impacts on ecosystems, communities, and economies.

In a focused study on the effects of droughts on public health, Salvador et al. [14] found that experiencing drought during pregnancy could have negative consequences on a child’s well-being, resulting in decreased stature and weight, especially among offspring of mothers with low education, low socioeconomic status, and residing in rural areas. Additionally, the study highlighted the association of droughts with decreases in food availability and alterations in household dietary patterns, potentially resulting in inadequate nutrition and heightened risks of premature birth and low birth weight among pregnant women.

Other extreme weather events reported in the literature that are associated with reproductive and maternal health include earthquakes. Aktoz et al. [17] explored the effects of these events on pregnancy outcomes and found that while no statistically significant differences were observed in preterm birth rates or the incidence of low-birth-weight infants among the studied groups, a significant increase in the proportion of infants classified as small-for-gestational-age emerged within the cohort affected by the seismic event.

All these events have significant impacts on women’s psychological well-being. The risk of prenatal mortality, premature birth and child development problems due to extreme heat temperatures creates additional stress for them. This psychological burden is intensified when considering the potential complications during pregnancy that could arise as a result of exposure to adverse weather conditions [30].

#### 3.1.3. Gender-Based Violence

The compiled literature underscores the intricate interplay between extreme weather events and gender-based violence (GBV) [10,11,19,23].

Logie et al. [19] explored this nexus, elucidating the complex pathways linking climate change-related factors to adverse sexual health outcomes and GBV. Employing an ecosocial approach, the authors constructed a conceptual framework delineating the mechanistic links between extreme weather events, HIV and sexually transmitted infection (STI) outcomes, and GBV. The study discerns that while climate change-related factors do not directly instigate GBV, they exacerbate pre-existing social disparities, such as gender inequity and LGBTQI+ marginalization, fostering environments conducive to GBV.

Similarly, Stone et al. [11] underscore the precarious conditions prevailing in post-disaster locales for women, where incidents of GBV surge in the aftermath of extreme weather events. Their review accentuates greater physical hazards in post-disaster situations, including increased gender-based violence, which further exacerbates women’s vulnerability to mental health problems like depression.

The forced displacement of populations, particularly women and children, increases the risk of sexual violence and exploitation. Neira et al. [20] observed that displacement often leaves vulnerable individuals with limited resources and uncertain legal status, leading some to resort to sex work to survive. Additionally, instances of kidnapping, rape, and sexual exploitation among the displaced escalate, increasing the prevalence of STIs and unintended pregnancies.

Rothschild and Haase’s review [27] underscores the exacerbation of domestic violence, sexual assaults, and homicides amid climate change-induced temperature rises and natural disasters. Studies consistently demonstrate that women are disproportionately affected by GBV during climate crises, often resulting in mental health disorders such as PTSD, depression, and anxiety.

#### 3.1.4. Occupational Health

Workers are also exposed to the effects of climate change, often for longer periods and with greater intensity than the general population. This exposure occurs because work activities end up “forcing” workers to put themselves in risky situations that other individuals could avoid, especially in outdoor activities such as agriculture and construction [34].

Spector et al. [12] did an exploratory review on the relationship between heat exposure and occupational injuries. Only two studies analyzed in this review have reported results by gender. One of them suggests both men and women frequently exposed to high temperatures at work have significantly higher risk of occupational injuries compared to those not exposed. The other one showed an increased risk of injuries with greater exposure to heat for males, including those under 25 years old and those who perform agricultural work. The authors hypothesize that the differences in the effect of heat on injuries according to gender and age are due to variations in gender distribution across industrial sectors and differences in age distribution according to level of experience, training, preventive behaviors, and physical exertion, respectively.

When analyzing the different climatic zones, Fatima et al. [22] found that the highest risk of occupational injuries during elevated temperatures was identified in humid subtropical climates, followed by oceanic climates and warm Mediterranean climates. Similarly, during heat waves, humid oceanic and subtropical climates presented the highest risk of workplace injuries. In this scenario, the authors noted that young male workers (under the age of 35) and those in industries such as agriculture, forestry, fishing, construction, and manufacturing were at high risk of workplace injuries during elevated temperatures. 

El Khayat et al. [15] carried out a scoping review to explore heat-related health effects among agricultural populations, identifying risk factors and preventative measures used to minimize heat stress exposure among farmworkers. The risk factors identified for both kidney disease and heat-related illnesses (HRIs) include gender, dehydration, heat strain, wearing inappropriate clothing, workload, piece-rate payment, job decision latitude, and hot environmental conditions. More specifically, the findings evidence that female farmers are more likely to suffer from heat-related illnesses than men, possibly due to lower water consumption and lack of access to sanitation facilities in agricultural settings. However, the segregation of agricultural tasks by gender contributes to men performing more strenuous tasks, thus increasing their risk of heat-related illness. Additionally, the reviewed studies suggest that female agricultural workers are at a greater risk of kidney disease as compared to men. However, two systematic reviews identified showed a positive association between males and a chronic disease of unknown etiology (CKDu).

#### 3.1.5. Health Issues Associated with Heat and Air Pollution

Three studies were identified that examine the impacts of air pollution and extreme heat on mortality and hospitalization rates.

Abed Al Ahad et al. [28] conducted an extensive review of 112 studies to assess the association between air pollution and mortality, as well as hospitalizations. They identified six major air pollutants (PM2.5, PM10, O_3_, CO, SO_2_, and NO_2_/NO_x_) correlated with an increase in mortality and hospitalization rates, particularly in cardiovascular, respiratory, and cerebrovascular diseases. Their findings suggest an inconsistency in how gender affects these outcomes, with females generally showing higher risks, but some studies indicating higher risks for males. Age may confound gender’s effect, with older females and males facing increased risks. This highlights the need to consider age and gender interactions in analyses to address potential confounding effects. 

In relation to exposure to extreme heat, Gifford et al. [26] conducted a systematic review that highlighted gender differences in the incidence and mortality associated with heat strokes. The results reveal higher rates of heat strokes in men compared to women, even after adjusting for age. This suggests a greater susceptibility of men to the effects of extreme heat on health.

Finally, providing a global insight into the impact of heatwaves on cardiovascular and respiratory health, Cheng et al.’s [24] systematic review and meta-analysis showed a higher risk of heart diseases in men during heatwaves, with a significant increase in the incidence of events such as heart disease and stroke. However, no significant associations were found between heatwaves and respiratory problems in men or women.

#### 3.1.6. Social Determinants and Climate Change

Climate change has extensive implications for human and public health, affecting diverse aspects of human well-being. In this regard, understanding the relationship between climate change, health, and gender is complex and multifaceted.

Factors such as such as age, gender, ethnicity, poverty, geographic location, socio-economic status, previous health conditions, and chronic diseases, among others, are intertwined and disproportionately affect certain population groups, exacerbating the risk of negative adverse events [20].

Several studies indicate that the impact of climate change differentially affects men and women, and that this phenomenon is largely attributable to existing disparities in gender roles, powers, and responsibilities in the domestic and community spheres [26,35,36]. Women often face a significant overload of housework and care tasks, while cultural and religious norms can limit their ability to make decisions in disaster situations. 

In low- and middle-income countries, the impacts of climate change are particularly severe due to their limited readiness and ability to address these climate-related risks. This vulnerability is compounded by fragile and under-resourced healthcare systems, as well as structural social inequities [37].

In this scenario, the review by Neira et al. [20] highlighted how water scarcity disproportionately affects women and girls, who often bear the responsibility of fetching water from distant sources. This task imposes physical strain, leading to injuries and hindering their ability to attend school. Additionally, adults may struggle to engage in income-generating activities due to the time spent on water collection, worsening financial instability in already marginalized households. This unequal distribution of responsibilities and resources can have profound implications for women’s health, increasing the risks of injury and psychological distress. Similarly, Dhimal et al. [18] reported findings regarding water scarcity in the Hindu Kush Himalayan Region, where the majority of women rely on the agriculture sector. 

These disparities extend to mental health outcomes. Mao and Agyapong [23] explored how social determinants contribute to mental health and resilience following both natural and man-made disasters. They examined factors such as gender, age, ethnicity, social support, and socioeconomic status in relation to survivors’ mental well-being. Their findings suggest that after disasters, adverse psychological consequences, such as PTSD and depression, tend to be more severe in female survivors, and panic and phobias tend to be more severe in children. Among migrants, for example, there is evidence of a high rate of psychological disorders and increased mental health symptoms due to decreased social support and contact. In relation to socioeconomic factors, the authors state that stress associated with insufficient resources can lead to emotional and behavioral consequences, and people with lower socioeconomic status may be more likely to have mental and physical problems compared to those of higher socioeconomic status after experiencing disasters. 

The occurrence of injuries or the loss of a family member due to climate change-related events can algo trigger emotional responses that contribute to the psychological burden associated with climate change. Coupled with the lack of solid social support or a support network to cope with these events, the impact on mental health can be further accentuated [10].

## 4. Discussion

In this scoping review we identified 19 studies addressing climate change, health, and gender. The search was carried out with a global scope, but there was a predominance of reviews that analyzed countries in North America, Europe, and Asia. No articles were found that studied Africa, and only three articles included two South American countries (Brazil and Chile) in their analyses [13,17,24]. This is significant because despite searching databases that include research from the Latin America and Caribbean regions, it is evident that these regions remain underrepresented in global research. This underrepresentation is crucial to note as issues do not manifest uniformly across countries.

The analysis of the included articles has yielded five key themes: mental health, reproductive health, gender-based violence, occupational health, and other health issues associated with heat and air pollution. Each theme sheds light on the differential effects of climate change on health, with distinct nuances and intersections.

The literature surveyed provides evidence of a differential effect on the mental health of men and women due to climate change [10,21,23,25,30]. Women face a higher risk of psychological impairment following extreme weather events, with increased rates of anxiety, depression, and PTSD. These mental health impacts may be due to a complex interaction of factors rooted in gender dynamics. In addition, women’s access to resources, including financial means and decision-making power, is often limited, which can exacerbate their susceptibility to adverse mental health outcomes. Socioeconomic disparities further compound these vulnerabilities, with marginalized women facing greater difficulties in accessing support systems and health services.

Climate change significantly affects pregnant women, who are at elevated risks of preterm birth, miscarriage, and stillbirth due to extreme heat exposure and environmental stressors. When these risks are combined with other vulnerabilities such as low education, poor socioeconomic status, and living in areas prone to extreme weather events, among others, health risks are even greater. These combined stressors not only threaten physical health but also put their mental health at significant risk [30], creating a complex web of challenges that must be addressed to protect this vulnerable group. 

In hot ambient or working environments, workers, especially those engaged in outdoor activities such as agriculture and construction, face prolonged and intense exposure to the effects of climate change, revealing differences between men and women. However, the results are not conclusive or definitive. This is because the reviewed studies [12,15,22] did not specifically aim to analyze gender differences in heat-related health effects and the associated risk factors among male and female workers. Given the distinct distribution of work and differing occupational exposures between men and women, it is essential to employ a gender-sensitive approach in research to identify gender-specific risk factors and better understand the differences in vulnerability to these conditions [15].

Some studies also highlight the impact of extreme weather events on the incidence of GBV, showing how the interplay of various factors exacerbates pre-existing social disparities. These include gender inequity, stigma, and marginalization, which together create conditions that increase the risk of GBV [19]. It is important to mention that only one review conducted an in-depth study on the incidence of GBV and climate change, exploring the underlying mechanisms and contextual factors that exacerbate this issue [19]. The remaining articles mention the connection between extreme weather events and GBV but do not focus on comprehensively understanding this relationship as it was not their objective. Gender data are generally difficult to collect, especially in developing countries where strong barriers persist, including cultural norms, lack of resources, and inadequate reporting systems. Having the data to produce knowledge on the subject is crucial to identifying research gaps and generating actions that can adequately address the problem.

The relationship between gender, age, mortality, and hospitalizations in the context of air pollution and high temperatures also presents inconsistent findings. Some studies reveal a higher risk of heat strokes and cardiovascular diseases in men during periods of intense heat. However, certain limitations were identified in the studies. For instance, one study points out deficiencies in age adjustment [28], while another indicates that, despite age adjustment, men have a higher mortality rate from heat strokes compared to women [26]. These discrepancies underscore the need to address confounding factors, such as age, when analyzing and interpreting the results.

This analysis of the literature clearly reveals that climate change does not affect everyone equally; rather, certain intersecting factors significantly increase the risk of experiencing adverse events. In addition to genetic factors, age, and gender, it is essential to consider the economies of the countries where individuals live and develop. Developing economies with low adaptive capacity risk experiencing greater impacts than developed economies. Having weak food, water, health, and infrastructure systems make them particularly vulnerable to climate change [38]. This limited readiness and ability to address climate-related risks could result in millions being driven into poverty.

This review of the literature also highlights the need for further study of intersectionality. The results primarily focus on women without specifying their ethnic origin, whether they are migrants, whether they are from the LGBTIQ+ community, or whether they are indigenous peoples, among other details. Disaggregating this information is important to evaluate the differential impacts based on intersectionality and to identify the most vulnerable groups.

Understanding gender and socioeconomic inequalities could help to achieve holistic and gender-sensitive approaches to climate change adaptation and mitigation strategies. Addressing these disparities requires targeted interventions that prioritize the specific needs of vulnerable populations, promote gender equity, and address structural barriers to resilience. By recognizing the differential impacts of climate change on health and the underlying social determinants, policymakers can develop more effective and inclusive responses to mitigate risks and build adaptive capacities within communities. 

## 5. Conclusions

Climate change impacts the lives and health of individuals, with its effects being particularly pronounced among populations in situations of greater vulnerability.

Through this scoping review, we were able to identify the differential effects of climate change on health according to gender. Furthermore, our findings elucidated how socio-economic and gender inequities intersect, exacerbating the risk of experiencing these effects. 

Future research is needed in the field of occupational health to study the differential exposures and health effects by gender, highlighting specific vulnerabilities. There is also a notable lack of long-term studies examining the health effects of climate change on women, particularly concerning the health consequences of air pollution and high temperatures. Future research should focus on these long-term effects to provide more precise insights to establish causal relationships between variables. Additionally, it is essential for future studies to include under-researched populations such as LGBTIQ+, indigenous communities, and migrants. By closing these knowledge gaps, it will be possible to advance more inclusive and effective climate interventions that recognize and respond to the diverse needs of all communities, ultimately fostering a more equitable and resilient society.

### Limitations

The present study has some limitations. The approach taken in this study involved analyzing the intersection of climate change, health, and gender from a global perspective, seeking to understand and describe the overall patterns and behaviors of the problematic. However, this broad approach may overlook the specificities of individual countries, potentially leading to an overgeneralization of the results. It is imperative to acknowledge the heterogeneity of climate-related challenges and adaptation strategies across different countries, which may result in divergent impacts and responses to climate change.

Lastly, it is important to note that no systematic assessment of the quality of the results from the meta-analyses included in our study was undertaken. The absence of this evaluation introduces a degree of uncertainty regarding the accuracy and precision of the synthesized evidence.

## Figures and Tables

**Table 1:** Search strategy used according to the database.

Database: PubMed		
Search	Strategy	Results
1	Climate Change[Mesh] OR Climate Change*[tiab] OR Climatic Change*[tiab] OR Global Warming[Mesh] OR Global Warm*[tiab] OR Sea Level Rise[Mesh] OR Sea-Level Ris*[tiab] OR Extreme Weather[Mesh] OR Extreme Cold[tiab] OR Extreme Heat[tiab] OR Cold Temperature[Mesh] OR Cold Temperature*[tiab] OR Hot Temperature[Mesh] OR Hot Temperature*[tiab] OR Droughts[Mesh] OR Tornado*[tiab] OR Tropical Storm*[tiab] OR Typhoon*[tiab] OR Hurricane*[tiab] OR Cyclon*[tiab] OR Natural Disasters[Mesh] OR Avalanche*[tiab] OR Earthquake*[tiab] OR Floods[tiab] OR Landslides[tiab] OR Tidal Wave*[tiab] OR Wildfire*[tiab] OR El Nino-Southern Oscillation[Mesh] OR El Nino[tiab] OR La Nina[tiab] OR Nino-Southern[tiab]	8602
2	Health[Mesh] OR Death[Mesh] OR Death*[tiab] OR Mortality[Mesh] OR Mortalit*[tiab] OR Wounds and Injuries[Mesh] OR Wounds[tiab] OR Injur*[tiab] OR Quality of Life [Mesh] OR “Quality Life”[tiab:~2] OR QoL[tiab]	196.473
3	Gender Identity[Mesh] OR Gender[tiab] OR Women[Mesh]	11.773
4	#1 AND #2 AND #3 and filters (Article type: Meta-Analysis, Review, Systematic Review, Publication date: 5 years)	34
Database: LILACS		
Search	Strategy	Results
1	(Climate Change$ OR Climatic Change$ OR Climate Change OR Global Warm$ OR Global Warming OR Global Drought OR Extreme Cold OR Extreme Heat OR Extreme Heat OR Cold Temperature OR Hot Temperature$ OR Droughts OR Drought$ OR Tornado$ OR Tropical Storm$ OR Tropical Storm OR Tropical Tempestade OR Typhoon$ Tifon$ OR Tufao OR Hurricane$ OR Hurricane$ OR Furacao OR Cyclon$ OR Cyclon$ OR Avalanch$ OR Earthquake$ OR Earthquake$ OR Flood$ OR Enchente$ OR Landslides OR Tidal Wave$ OR Maremoto$ OR Wildfire$ OR Incendio$) AND (Health OR Salud OR Saude OR Death$ OR Mortali$ OR Wounds OR Injur$ OR Herid$ OR Quality Life OR QoL OR Calidad de Vida OR Qualidade de Vida) AND (Gender OR Genero)	23
Database: SciELO		
Search	Strategy	Results
1	Climate Change* OR Climatic Change* OR Climate Change OR Global Warm* OR Global Warming OR Global Drought OR Sea-Level Ris* OR Extreme Cold OR Extreme Heat OR Extreme Heat OR Drought OR Tornado* OR Tropical Storm* OR Tropical Storm OR Tropical Storm OR Tropical Storm OR Typhoon* OR Typhoon* OR Tufao OR Hurricane* OR Hurricane* OR Fracao OR Cyclon* OR Cyclon* OR Natural Disasters OR Avalanch* OR Earthquake* OR Earthquake OR Earthquake OR Floods OR Inundacion* OR inundações OR Landslides OR Tidal Wave* OR Maremoto* OR Wildfire* OR Incendio* OR “El Niño” OR “La Niña”) AND (Genero OR Gender OR Women OR Mujer)	15

**Table 2:** Characteristics of studies included in the review (n = 19).

Author, Year, and Reference	Study Type	Region	Study Period	Number of Articles Included in the Review	Aim	Analyzed Population
Ramadan and Ataallah (2021) [10]	Review	Global	Not specified	No information	To understand the currently, scientifically proven impact of climate change-related disasters on mental health and understanding the different methods of solving the problem at the corporate level, by trying to decrease greenhouse gas emissions to zero, and at the individual level by learning how to cope with the impacts of those disasters.	Not specified
Stone et al. (2022) [11]	Scoping review	Global	2007 to 2020	20	To understand what is known from the existing literature regarding the mental health impacts of climate change on women.	Women, young women, girls, gender non-binary femme-identified people, and Two-Spirit people
Spector et al. (2019) [12]	Review	North America (US and Canada), Europe (Italy and Spain), Asia (Thailand and China), and Australia	Not specified	12	To review the emerging literature on the relationship between heat exposure and occupational traumatic injuries, and to discuss implications of this work.	All workers, workers in all industries, outdoor construction workers, agricultural workers, Thai Cohort Study participants who reported working for income, and aluminum smelter workers
Huang et al. (2023) [13]	Systematic review and meta-analysis	North America (US), South America (Brazil), Australia, and Asia (China, India, Japan, the Philippines, and Republic of Korea)	Inception to April 2022	71	To provide a systematic review with meta- analysis of current evidence on the risks of all reported health outcomes related to cyclones, and to identify research gaps and make recommendations for further research.	Population-based studies with no restriction on sex, age, or region
Salvador et al. (2023) [14]	Review	Global	Not specified	No information	To analyze the observed and projected trends in drought occurrence, assess the main impacts of drought on health, and provide an overview of prevention and adaptation policies, providing recommendations to address future threats of climate change and to enhance resilience.	Not specified
El Khayat et al. (2022) [15]	Review	Global	Inception to December 2021	92	To summarize the available evidence on the effects of climate change on farmworkers’ health with a focus on heat-related illnesses. To identify the risk factors for heat-related illnesses among farmworkers, and to review the preventive measures that are used to minimize heat stress exposure among farmworkers.	Farmworkers of all age groups
Rekha et al. (2023) [16]	Systematic review	North America (US and Canada), Europe (Germany, Spain, and Sweden), Australia, and Asia (China, Israel, and Bangladesh)	Inception to December 2021	23	To compile all known evidence-based research on the effects of heat stress on Adverse Pregnancy Outcomes (APO)	Treated pregnant women and newborns
Aktoz et al. (2023) [17]	Systematic review and meta-analysis	Asia (China and Iran), South America (Chile), and Australia (New Zealand)	Inception to April 2023	5	To evaluate the available evidence, estimate the overall effect, and identify key research gaps about the effect of earthquakes on pregnant women.	Pregnant women
Dhimal et al. (2021) [18]	Narrative review	Asia	Not specified	No information	To document how climate change has impacted and will impact the health and well-being of the people in the Hindu Kush Himalayan region, and to offer adaptation and mitigation measures to reduce the impacts of climate change on the health and well-being of these people.	Population of the Hindu Kush Himalayan region
Logie et al. (2024) [19]	Scoping review	Global	Without time restrictions	108	To examine the literature on associations between climate change and sexual health.	Not specified
Neira et al. (2023) [20]	Literature review	Middle East	2000 onwards	No information	To synthesize current knowledge on the effects of climate change on the health of people living in the Eastern Mediterranean and Middle East (EMME) region, with a particular focus on exposure to extreme heat, water shortages, air pollution, vector-borne diseases, and the health of displaced populations.	Not specified
Adu et al. (2023) [21]	Literature review	North America (US and Canada), Europe (Portugal), and Asia (Thailand)	2019 to 2022	8	To synthesize the currently available literature regarding the impact of wildfire on mental health, specifically the psychological reactions of children to wildfires.	≤20 years old
Fatima et al. (2021) [22]	Systematic review	North America (US and Canada), Europe (Italy and Spain), Asia (China), and Australia	Inception to July 2020	24	To summarize the existing epidemiological evidence on the impact of extreme heat (hot temperatures and heatwaves (HW)) on occupational injuries (OI) in different climate zones, and to assess the individual risk factors associated with workers and workplaces that contribute to heat-associated OI risks.	Non-military workers
Mao and Agyapong (2021) [23]	Literature review	Not specified	Not specified	21	To explore the vulnerability and protective social determinant factors which have an impact on the mental health and resiliency in survivors of disasters.	Not specified
Cheng et al. (2019) [24]	Systematic review and meta-analysis	North America (US), South America (Brazil), Australia, Asia (China, the Philippines, Republic of Korea, Thailand, Vietnam, Iran, and Russia), and Europe (Spain, Germany, France, Slovenia, The Netherlands, and Russia)	Inception to November 2018	54	To quantify heatwave effects on four major health outcomes: cardiovascular and respiratory morbidity and mortality.	Not specified
Chique et al. (2021) [25]	Literature review	Global	1980 onwards	59	To collate, integrate, and analyze psychological morbidity data from populations exposed to Extreme Weather Events.	General population
Gifford et al. (2019) [26]	Systematic review and meta-analysis	North America (US and Canada), Europe (UK), Asia (China, Korea, and Japan), and Australia	Without time restrictions	36	To determine the relative risk of heat illness in women compared with men through an exhaustive literature review and meta-analysis.	Adult or adolescent human data reporting comparable male and female heat illness rates
Rothschild and Haase (2022) [27]	Narrative review	Not specified	Not specified	No information	To provide an overview of the impacts of climate change on women’s mental health.	Women
Abed Al Ahad et al. (2020) [28]	Scoping review	Europe	2012 to 2020	112	To provide a comprehensive review and narrative summary of the literature on the association between air pollution and weather with mortality and hospital admissions.	Not specified

**Table 3:** Summary of key results by theme.

Theme	Summary of the Impacts of Climate Change on Health According to Gender and Considering the Social Determinants of Health
Mental Health	Risk factors: magnitude of disaster, female gender, younger age, low education and socioeconomic status, loss or injury of loved ones, minority or ethnic status, immigrant groups, indigenous people, family instability, intimate partner violence, pre-existing health conditions or/and mental health issues, and lack of access to health and support services [10,11,21]. Women experience a wide range of emotions due to climate change, with some experiencing specific psychological disorders such as anxiety, major depressive disorder (MMD), and post-traumatic stress disorder (PTSD) [11,13,23,25]. There is a higher prevalence of psychological disorders among women linked to traditional gender roles and existing inequalities [23].
Occupational Health	Both men and women exposed to high temperatures at work have a significantly higher risk of occupational injuries compared to those not exposed [12]. Health risk factors for farmworkers include gender, dehydration, heat strain, inappropriate clothing, workload, piece-rate payment, job decision latitude, and hot environmental conditions [15]. Piece-rate payment and years working in agriculture were identified as risk factors for kidney disease among women [15]. Pregnant working women, migrants, and child farmworkers are especially vulnerable groups [15,16].
Reproductive Health	Exposures to heat, air pollution, food insecurity, and vector-borne diseases have direct and significant effects on reproductive and maternal health [20]. There is a strong association between heat stress and adverse pregnancy outcomes including miscarriages, premature birth, stillbirth, low birthweight, and congenital abnormalities [16]. Child-bearing women face increased risks during displacement due to inadequate hygiene, nutrition, and antenatal/postnatal care. This increases the chances of reproductive tract infections and malnourishment, and together with a lack of access to medical services, raises the risk of maternal morbidity and mortality, preterm birth, miscarriage, stillbirth, and other perinatal and neonatal issues [14,20]. Drought impacts maternal and reproductive health by reducing food supply and increasing food prices, leading to malnutrition and lower dietary diversity, particularly affecting farmers, pregnant women, children under five, the elderly, and low socioeconomic groups. This exacerbates the risks of adverse pregnancy outcomes [14].
Gender-based violence	Climate change-related factors do not directly cause gender-based violence (GBV); rather, they exacerbate existing social inequities, creating environments that enable GBV to occur [19,27]. There are multiple pathways between Extreme Weather Events and GBV, including structural, community, interpersonal, and intrapersonal levels, among others [19]. Displacement due to climate change further elevates the risk of sexual violence and exploitation for women and children [11,19,20].
Other health issues	The findings indicate inconsistency in gender effects in relation to health issues associated with extreme heat and air pollution, with females generally showing higher risks, but some studies also report higher risks for males [24,26,28]. Age may confound the effect of gender, with older females and males facing increased risks [28].

## Data Availability

No new data were created or analyzed in this study. Data sharing is not applicable to this article.
